# The High Five Model as a predictor of academic performance over conventional psychological predictors in university students

**DOI:** 10.3389/fpsyg.2024.1383154

**Published:** 2024-05-22

**Authors:** Jessica V. Quito-Calle, Alejandro César Cosentino

**Affiliations:** ^1^Psychology Research Group (GIPSI-SIB), Faculty of Psychology, Universidad Politécnica Salesiana, Cuenca, Ecuador; ^2^Department of Psychology, Faculty of Social Sciences, Universidad de Palermo, Buenos Aires, Argentina

**Keywords:** High Five Model, factors of the Five Highs Model, academic performance, academic motivation, test anxiety, academic procrastination

## Abstract

**Introduction:**

The High Five Model (HFM) categorizes five positive human characteristics-erudition, peace, joviality, honesty, and tenacity-utilizing an inductive psycholexic approach. This study examines the predictive power of HFM on academic performance among university students, hypothesizing that it surpasses conventional predictors such as academic motivation, exam anxiety, and academic procrastination.

**Methods:**

A non-experimental cross-sectional correlational design was implemented using a non-probabilistic convenience sample of 1,007 Ecuadorian university students (403 females). Self-reported measures of the “high factors,” academic motivation, exam anxiety, and academic procrastination were collected. Linear regression analysis was utilized to evaluate the predictive capacity of the HFM on academic performance.

**Results:**

The analysis revealed that the high factors of the HFM significantly predict academic performance, demonstrating a stronger predictive ability than traditional psychological predictors.

**Discussion:**

The findings suggest that incorporating the HFM into academic settings could enhance understanding and prediction of student performance. This could potentially inform targeted interventions that leverage these high factors, thereby fostering better academic outcomes. Further research could explore the integration of the HFM with other educational strategies and its applicability across diverse educational contexts.

## 1 Introduction

Positive personality traits are a domain explored by Positive Psychology, which, from a scientific perspective, examines how individuals distinguish themselves from each other based on their human strengths and virtues (Sheldon and King, 2001; Tintaya Condori, 2019). Models of individual and moral positive personality traits have been studied for thousands of years, both within religious and philosophical traditions of both Eastern and Western cultures (Cosentino, 2010), and it is from the 20th century onwards that this subject became a matter of interest in Psychology. Particularly, the High Five Model (HFM) proposed by (Cosentino and Castro Solano, 2017) is a model that effectively identifies positive human characteristics. The HFM is a factorial model of individual positive traits based on an inductive psycholexic approach. It comprises the positive human factors known as “high factors”: erudition, peace, joviality, honesty, and tenacity. These factors differ from the factors in the Big Five Model (BFM) of normal personality, despite their relationships. In this context, HFM factors can be considered as the positive poles of BFM. Castro Solano and Cosentino (2019) posit that the positive traits or high factors of the HFM are relatively stable within each individual and are represented by positive psychological characteristics. Moreover, the high factors of the HFM possess certain notable attributes: they can be measured, vary among individuals, and may increase or decrease due to internal or external influences.

Despite the limited number of studies on the HFM due to its recent proposal, its results are robust as it has been reasonably linked to numerous variables. Among its findings, it has been observed that HFM factors, as positive personality traits, are negatively associated with health indicators and positively correlated with emotional, psychological, and social wellbeing. (Castro Solano and Cosentino, 2019) have also demonstrated HFM's association with elements of the academic context and academic performance, utilizing self-reported values by students for performance analysis. In their study, it was found that the high factors of tenacity and erudition were the only ones positively associated with academic performance. Therefore, the objective was set to demonstrate that the HFM factors not only predict academic performance but also retain their predictive power beyond the contribution of common psychological predictors such as academic motivation (Baker, 2004; Kaufman et al., 2008; Petersen et al., 2009; Sommer and Dumont, 2011; Bailey and Phillips, 2016; Orbegoso, 2016; Deng and Shi, 2023), exam anxiety (Rana and Mahmood, 2010; Ávila Toscano et al., 2011; Piemontesi and Heredia, 2011; Sikhwari, 2014; Lomelí-Parga et al., 2016; Owan, 2020; Manchado Porras and Herví Ortega, 2021), and academic procrastination (Sánchez-Hernández, 2010; Furlan et al., 2012; Delgadová and Gullerová, 2015; Kim and Seo, 2015; Jones and Blankenship, 2021). Furthermore, this study utilized institutional academic performance records, an objective measure, instead of the subjective self-reported measure used by Castro Solano and Cosentino (2019). Findings of this study contribute to Educational Psychology and Positive Psychology by providing a broader conceptual framework for the HFM to relate to academic performance, advancing the boundaries of knowledge in these fields.

Current paper is structured as follows. Section 2 elucidates the HFM and its constituent factors. Subsequently, it addresses Academic Motivation and its dimensions, followed by an examination of Exam Anxiety and its dimensions. Finally, it delves into Academic Procrastination. In Section 3, a literature review is presented, where theoretical foundations and empirical findings related to the study variables are compiled, analyzed, and compared. Section 4 establishes the predictors as dimensions of Academic Motivation, Exam Anxiety, and Academic Procrastination, with Academic Performance serving as the outcome variable. The goal is to measure relationships and causal relationships, and five hypotheses are introduced. Section 5 provides details about the study participants, the instruments employed, and the procedure for hypothesis testing. Section 6 presents the empirical findings of the experiment, including the testing of the proposed theoretical model through linear regression analysis. In Section 7, results presented in previous sections are discussed in conjunction with other empirical studies in the field. Finally, Section 8 offers conclusions related to the achievement of the study's objective and suggests potential avenues for future research.

## 2 Background

The HFM was developed by Cosentino and Castro Solano (2017) based on the identification of positive human characteristics from the perspective of ordinary individuals, irrespective of their moral nature. To construct this model of socially shared positive individual traits, authors decided to create the High Five Inventory (HFI), comprising 23 items distributed across five subscales of socially shared positive human characteristics. The HFM was developed with a deeply inductive psycholexic approach, adopting statistical and syntactic criteria instead of semantic selection criteria. This was done in an effort to exclude any theoretical influence from an academic standpoint, with the aim of achieving replication across different populations (Castro Solano and Cosentino, 2019). In this manner, (Cosentino and Castro Solano, 2017) created a model of socially shared positive human trait factors that could potentially be replicated in other samples. The model's five positive factors of the HFM—erudition, peace, joy, honesty, and tenacity–maintain a conceptual and empirical proximity to the factors of Big Five Model (BFM): extraversion, agreeableness, conscientiousness, neuroticism, and openness to experience. However, even though a relationship exists between the two models, the high factors of the HFM are considered the positive poles of the BFM factors, but they are not mere duplications or repetitions of BFM factors (Cosentino and Castro Solano, 2017). The HFM factors are relatively stable within each individual and are represented by positive psychological characteristics. Furthermore, they possess several significant attributes: (a) they can be measured, (b) they vary among individuals, and (c) they may increase or decrease due to internal or external influences (Castro Solano and Cosentino, 2019).

Term “high” for the factors in the HFM was selected for two main reasons. Firstly, to signify that these factors are linked to the individual characteristics of ordinary individuals who highly value or regard them positively. Secondly, they are considered positive poles, in relative terms, of the factors in BFM (Castro Solano and Cosentino, 2019). The high factors of the HFM are positively associated with the Big Five factors. In this manner, the high factor Erudition is positively associated with the Openness to Experience factor; the high factor Peace is associated with Emotional Stability (in contrast to Neuroticism); the high factor Joviality is linked with Extraversion; the high factor Honesty is associated with Agreeableness, and the high factor Tenacity is associated with Conscientiousness (Cosentino and Castro Solano, 2017).

As a result, personality traits, through motivation, explain the distinct behaviors of each individual in various situations (Costa, 1992; Ariani, 2013). Therefore, if these traits are not the source of formation, motivation for learning is affected by contextual factors and, above all, by personality (Colquitt et al., 2000; Ariani, 2013). It is appropriate to consider these differences when exploring individual performance in various areas, including academics. Authors like Salgado (1997) assert that conscientiousness and neuroticism are predictors of performance and motivation. Motivation is an internal state that drives people to take actions directed toward goals. This motivation influences the type of strategy employed to carry out an action and the time and perseverance invested in achieving it. When referring to an internal state, it implies that it is individual, dependent on the individual and the specific circumstances they are experiencing. Therefore, it could be said that this motivation largely depends on an individual's personality trait (Fuertes et al., 2020). Motivation can be expressed through a continuum of self-determination with three fundamental positions reflecting the degree of autonomy on which behaviors are based: amotivation, extrinsic motivation, and intrinsic motivation (Stover et al., 2014). Amotivation implies that a person acts without the intention of achieving a result because they do not see any connection between their actions and the consequences of those actions. It is characterized by the individual's perception of a lack of control over events, a sense of incompetence, and the absence of purposes (Sum et al., 2022). Extrinsic motivation refers to performing an activity to achieve a specific outcome or obtain a reward. The subject's broad range of behaviors leads them to carry out actions with the aim of obtaining an expected result, rather than for the inherent reasons behind that action (Deci and Ryan, 2004). Extrinsic motivation can be further categorized into four subtypes of progressive regulation: external, introjected, identified, and integrated. Intrinsic motivation occurs when an individual engages in an activity for the pleasure and enjoyment it provides because it is inherently pleasant or interesting. Therefore, what drives them to act is the activity itself as an end in itself. Intrinsic motivation can be divided into three subtypes commonly referenced in the academic context: knowledge orientation, achievement orientation, and experience orientation.

Simultaneously, individuals who do not strongly experience this need for achievement motivation tend to respond with avoidance-oriented emotions, such as anxiety, defensiveness, and fear of failure (Ferrari and Scher, 2000; Bryce, 2003; Krispenz et al., 2019). Anxiety, in addition to being an emotional reaction, is also a personality factor. Like any other emotion, anxiety involves at least three components or response systems: cognitive, physiological, and behavioral. The activation of these anxiety components can lead to a higher error rate or hinder our performance, significantly deteriorating our achievement motivation. This may lead us to resort to easier and more accessible tasks or trigger an escape or avoidance response from a risky situation, potentially affecting us, especially at a psychological level, causing anxiety (Sandín and Chorot, 1995). In terms of affective response, exam anxiety is associated with unpleasant feelings of agitation, insecurity, and helplessness, which can have motivational consequences, such as avoidance tendencies in academic performance-related contexts (Thomas and Cassady, 2019). Hodapp (Hodapp et al., 1995) proposes a model comprised of four dimensions grouped into a first-order factor of Exam Anxiety. These dimensions include the affective component: emotional reactivity; and the cognitive components: worry, lack of confidence, and cognitive interference. Emotional reactivity involves all autonomic reactions that tend to occur under exam-related stress (Liebert and Morris, 1967). It refers to the perception of affective or physiological arousal experienced by the student during the evaluation (Hodapp, 1996). Worry is identified as any expression of concern about one's own performance, consisting of recurring thoughts about the personal or social consequences of potential poor performance in an exam. Lack of confidence entails negative beliefs about one's ability to perform adequately on the exam. Cognitive interference refers to cognitions that interfere in test situations, regardless of their specific content. These are thoughts that lead to distraction and cognitive blockage (Hodapp et al., 1995).

Current studies, such as those by (Rahardjo et al., 2013), propose that individuals with higher levels of anxiety tend to be more prone to procrastination with the aim of avoiding aversive conditions or unpleasant states (Rothblum et al., 1986; Schouwenburg, 1995; Ferrari and Scher, 2000; Scher and Osterman, 2002; Van Eerde, 2003; Onwuegbuzie, 2004; Rosário et al., 2007; Rahardjo et al., 2013). Consequently, individuals with higher anxiety fear poor evaluation, fear not meeting expectations, and fear social disapproval, resulting in the behavior of postponing tasks that involve interacting with people (Furlan et al., 2012). A wide range of research has supported the premise that personality is an integral part of academic procrastination (Johnson and Bloom, 1995; Senécal et al., 2003; Lee et al., 2006; Özer et al., 2009; Çam, 2013; Doğan et al., 2014). Academic procrastination is the prevalent tendency to delay the academic process and related activities, often associated with anxiety. It is a relatively common phenomenon among university students (Imig, 2020), characterized by a behavior that involves postponing a task and, instead, engaging in less important tasks, avoiding responsibilities and commitments, ultimately affecting academic performance (Estremadoiro Parada and Schulmeyer, 2021). Moreover, procrastination is viewed as a widespread tendency to defer the start and/or completion of planned tasks within a specified timeframe. This inclination toward postponement is often accompanied by subjective distress and is not merely a matter of low responsibility and time management but represents a genuine issue of self-regulation on cognitive, emotional, and behavioral levels (Klingsieck, 2013). The propensity to procrastinate can be exacerbated by factors such as perceived self-competence (Haghbin et al., 2012), low self-control (Uzun et al., 2020), fear of failure (Zhang et al., 2018), depression (Kınık and Odacı, 2020), low self-esteem (Hajloo, 2014), or anxiety (Spada et al., 2006), among others.

## 3 Related works

O'Connor and Paunonen (2007), Vedel (2014), Lamas (2015), Sorić et al. (2017), Stajkovic et al. (2018), Hidalgo-Fuentes et al. (2021) conducted studies on the relationship between the Five Factor Model of personality and academic performance. Sorić et al. (2017), Stajkovic et al. (2018) utilized subjective measures, such as self-report, for the analysis of academic performance, while O'Connor and Paunonen (2007), Vedel (2014), Lamas (2015), Sorić et al. (2017), Stajkovic et al. (2018), Hidalgo-Fuentes et al. (2021) employed objective measures, such as the institution's recorded grade point average, for the analysis of academic performance. The authors (O'Connor and Paunonen, 2007; Vedel, 2014; Lamas, 2015; Sorić et al., 2017; Stajkovic et al., 2018; Hidalgo-Fuentes et al., 2021), consistently found that the factor of conscientiousness (known as tenacity in the HFM) is frequently associated with academic performance and serves as a powerful, significant predictor thereof. Lamas (2015) discovered a positive relationship between the personality traits of openness and conscientiousness and the academic performance of university students. Finally, Castro Solano and Cosentino (2019) found that high levels of tenacity and erudition are positively associated with academic performance.

Komarraju et al. (2009), Vallerand et al. (1992), Clark and Schroth (2010), Hazrati-Viari et al. (2012), De Feyter et al. (2012) examined the relationship between the Big Five personality traits and academic motivation. They found that conscientiousness and openness to experience are positively associated with intrinsic motivation, while extraversion, conscientiousness, and neuroticism are positively related to extrinsic motivation. Conscientiousness and agreeableness are positively related to amotivation. However, Clark and Schroth (2010) consider openness to experience as a key personality factor predicting academic motivation. Moreover, individuals who are intrinsically motivated to attend university tend to be extroverted, agreeable, conscientious, and open to new experiences. De Feyter et al. (2012) noted a significant positive correlation between agreeableness and conscientiousness and academic motivation. On the other hand, Baker (2004), Kaufman et al. (2008), Bailey and Phillips (2016), Petersen et al. (2009), Sommer and Dumont (2011), Deng and Shi (2023) explored the relationship between academic motivation and academic performance. Baker (2004), Kaufman et al. (2008), Sommer and Dumont (2011), Deng and Shi (2023) relied on objective measures, such as the institution's recorded grade point average, for the analysis of academic performance, while Bailey and Phillips (2016), Petersen et al. (2009) used subjective measures, such as self-reports. Baker (2004), Kaufman et al. (2008), Bailey and Phillips (2016), Peterson and Seligman (2004), Sommer and Dumont (2011), Deng and Shi (2023) found that intrinsic motivation predicted better academic performance, particularly knowledge-oriented intrinsic motivation being the only significant predictor of academic performance. Amotivation, on the other hand, predicted lower academic performance, while extrinsic motivation showed no significant relationship with academic performance. Kaufman et al. (2008) found that intrinsic motivation and extrinsic motivation predicted higher and lower grades, respectively. Peterson and Seligman (2004), Deng and Shi (2023) found that extrinsically regulated external motivation negatively predicted academic performance in university students. Deng and Shi (2023) also found that intrinsically regulated introjected motivation significantly predicted academic performance. Lastly, Sommer and Dumont (2011) observed a positive impact of extrinsic motivation on academic performance.

Francisquelo and Furlan (2015), Diav et al. (2017), Márquez et al. (2008), O'Connor and Paunonen (2007), McIlroy and Bunting (2002), Poole (2014), Curelaru and Diac (2021), Lim and Ortiz-bance (2013) investigated the relationship between exam anxiety and the Big Five personality traits in university students. Francisquelo and Furlan (2015), Márquez et al. (2008), Curelaru and Diac (2021), Lim and Ortiz-bance (2013) found that neuroticism is the predictor for the emergence of exam anxiety, especially in the cognitive dimensions (interference, worry, lack of confidence) and in the emotional dimension, while conscientiousness plays a complementary role in the opposite direction. Diav et al. (2017), Lim and Ortiz-bance (2013) consider the personality trait openness to experience to have a negative association with worry and lack of confidence, indicating that higher openness to experience is related to less concern about negative consequences and greater self-confidence. Márquez et al. (2008) found negative and significant relationships between the factors of openness and agreeableness with exam anxiety and positive correlations with the factor neuroticism (McIlroy and Bunting, 2002; O'Connor and Paunonen, 2007; Poole, 2014).

Curelaru and Diac (2021) found that low scores in extraversion, agreeableness, conscientiousness, and openness to experience promote high levels of exam anxiety, especially in the cognitive domain. On the other hand, Piemontesi et al. (2012), Ávila Toscano et al. (2011), Manchado Porras and Herví Ortega (2021), Rana and Mahmood (2010), Sikhwari (2014), Owan (2020) studied the relationship between exam anxiety and academic performance based on a subjective measure such as self-report. Piemontesi and Heredia (2011), Ávila Toscano et al. (2011), Rana and Mahmood (2010), Sikhwari (2014), Owan (2020) found that the interference dimension was moderately negatively correlated, while lack of confidence also showed a weak negative correlation with academic performance. However, worry and emotional factors did not demonstrate a significant relationship with academic performance. Manchado Porras and Herví Ortega (2021) found that higher subjective perception of physiological activation (emotional intensity) and more intrusive or irrelevant thoughts that distract from the task (interference) led to lower academic performance. In other words, exam anxiety has a negative relationship with academic performance.

Saraswati (2017), Ocansey et al. (2022), Börekci (2022), Shaw and Choi (2022), Kim et al. (2017) examined the relationship between the Big Five personality traits and academic procrastination. Saraswati (2017), Ocansey et al. (2022), Börekci (2022) found that extraversion, conscientiousness, and neuroticism emerged as predictors of procrastination tendencies among students, while Ocansey et al. (2022), Börekci (2022), Shaw and Choi (2022), Kim et al. (2017) stated that academic procrastination is negatively associated with openness to experience, conscientiousness, extraversion, and agreeableness, but positively related to neuroticism. Neuroticism is the strongest predictor of academic procrastination, followed by openness to experience. On the other hand, Kim and Seo (2015), Furlan (2013), Lomelí-Parga et al. (2016), Jones and Blankenship (2021) examined the relationship between academic procrastination and academic performance. Kim and Seo (2015), Furlan et al. (2012), Lomelí-Parga et al. (2016) relied on a subjective measure, such as self-report, for the analysis of academic performance, while Jones and Blankenship (2021) used an objective measure, such as the institution's recorded grade point average, for the analysis of academic performance. All authors concurred that academic procrastination is negatively correlated with academic performance.

Based on the studies presented, it is important to take as a starting point the latest publication by Castro Solano and Cosentino (2019), where the HFM is linked to elements of the academic context, including academic performance. In their analysis of academic performance, they relied on subjective measures, specifically self-reported values. This limitation was identified in the study by Castro Solano and Cosentino (2019). Consequently, they did not utilize objective measures obtained directly from institutional records, which could serve as a more reliable method for the study (Castro Solano and Cosentino, 2019). In the present study, it was demonstrated that the high factors of the HFM are predictors of academic performance, and for the analysis of academic performance, objective measures obtained directly from institutional records were used. Furthermore, the HFM factors were included in the analysis to demonstrate that they can go beyond the typical psychological predictive models associated with academic performance. Among the typical predictors are academic motivation, exam anxiety, and academic procrastination, highlighting the novelty in the proposed research.

## 4 Present study

Despite the relatively short history of research publications due to the novelty of the HFM, it has proven to be a suitable model as it surpasses the usual capacity of personality measurement to predict its connection to many variables in a reasonable manner, particularly with academic performance (Lounsbury et al., 2009; Cosentino and Solano, 2012). In the latest study by Castro Solano and Cosentino (2019), the model is linked to academic performance. For their analysis, subjective data were used, as it involved self-reported values provided by the students. The findings revealed that the high factors, namely, tenacity and erudition, were the only ones positively associated with academic performance.

In line with the studies mentioned above, the proposal aims to determine which of the MCA factors predict academic performance above the usual predictors such as academic motivation, exam anxiety, and academic procrastination. To accomplish this, in contrast to the study conducted by Castro Solano and Cosentino (2019), the present study uses an objective measure for the analysis of academic performance, specifically, using grades from institutional records.

In this context, the dimensions of Academic Motivation, Test Anxiety, and Academic Procrastination are established as outcome variable, with Academic Performance as the outcome variable, aiming to assess relationships and causalities. Consequently, five hypotheses are posited:

**Hypotheses 1**. *High levels of the HFM predict academic performance*.

Hypothesis 1 is proposed to verify the assertion made by Castro Solano and Cosentino (2019) that high levels of Academic Motivation predict academic performance.

**Hypotheses 2**. *High levels of the HFM factors enhance the prediction of academic performance compared to the dimensions of academic motivation*.

Hypothesis 2 is proposed to verify the assertion made by Castro Solano and Cosentino (2019) that high levels of Academic Motivation factors improve the prediction of academic performance compared to the dimensions of academic motivation (Baker, 2004; Kaufman et al., 2008; Petersen et al., 2009; Sommer and Dumont, 2011; Bailey and Phillips, 2016; Orbegoso, 2016; Deng and Shi, 2023).

**Hypotheses 3**. *High levels of the HFM factors improve the prediction of academic performance compared to dimensions of test anxiety*.

Hypothesis 3 is proposed to verify the assertion made by Castro Solano and Cosentino (2019) that high levels of Academic Motivation factors improve the prediction of academic performance compared to dimensions of test anxiety (Rana and Mahmood, 2010; Ávila Toscano et al., 2011; Piemontesi and Heredia, 2011; Sikhwari, 2014; Byrne, 2016; Owan, 2020; Manchado Porras and Herví Ortega, 2021).

**Hypotheses 4**. *High levels of the HFM factors improve the prediction of academic performance compared to academic procrastination*.

Hypothesis 4 is proposed to verify the assertion made by Castro Solano and Cosentino (2019) that high levels of HFM factors improve the prediction of academic performance compared to academic procrastination (Sánchez-Hernández, 2010; Furlan et al., 2012; Kim and Seo, 2015; Lamas, 2015; Jones and Blankenship, 2021).

**Hypotheses 5**. *High levels of the HFM factors improve the prediction of Academic Performance beyond the capacity of the usual predictors, including Academic Motivation, Test Anxiety, and Academic Procrastination*.

Hypothesis 5 is proposed to verify the assertion made by Castro Solano and Cosentino (2019) that high levels of Academic Motivation factors improve the prediction of Academic Performance beyond the capacity of the usual predictors, such as Academic Motivation (Baker, 2004; Kaufman et al., 2008; Petersen et al., 2009; Sommer and Dumont, 2011; Bailey and Phillips, 2016; Orbegoso, 2016; Deng and Shi, 2023), Test Anxiety (Rana and Mahmood, 2010; Ávila Toscano et al., 2011; Piemontesi and Heredia, 2011; Sikhwari, 2014; Byrne, 2016; Owan, 2020; Manchado Porras and Herví Ortega, 2021), and Academic Procrastination (Sánchez-Hernández, 2010; Furlan et al., 2012; Kim and Seo, 2015; Lamas, 2015; Jones and Blankenship, 2021).

## 5 Materials and methods

### 5.1 Participants

A total of 2,765 university students were included in the study, of whom 2,667 agreed to participate voluntarily, while 98 declined the invitation. Strict criteria were applied for sample selection: participants had to be enrolled in the day section and have continuity in their courses. Those under 18 years of age, enrolled in the night section, with a history of failing courses in any academic period, as well as those presenting mental disorders, learning difficulties, or intellectual disabilities that could impair their ability to respond to the test were excluded.

A total of 1,007 participants took part in the study, including 403 women (39.0%). Their average age was 21.88 years (SD = 3.69 years), with the majority falling within the 18-19 age group (42.1%). These participants belonged to various faculties within a private university in Ecuador, categorized as follows: Social Sciences and Humanities (*n* = 285, 28.3%), Science and Technology (*n* = 399, 39.6%), Business and Economics (*n* = 158, 15.7%), Life Sciences (*n* = 113, 11.2%), and Education (*n* = 52, 5.2%). The sampling method employed was non-probabilistic by convenience, with voluntary, informed, and anonymous participation. No financial compensation was provided for participation.

Groups distribution according to participants' sociodemographic profile is outlined in Table 1, while the breakdown of the Faculties and Careers to which the participants are affiliated is presented in Table 2.

**Table 1:** Sociodemographic description of study participants.

**Variable**	**Category**	**Quantity**	**Percentage**
Gender	Male	604	60.1%
	Female	403	39.9%
Age (years)	18 - 20	424	42.1%
	21–23	335	33.3%
	24–26	164	16.3%
	27 or more	84	8.3%
Marital status	Single	962	95.5%
	Married	23	2.3%
	Divorced	3	0.3%
	Separated	4	0.4%
	Common-law marriage	14	1.4%
	Widowed	1	0.1%
Degree program semester	First (1st semester of 1st year)	136	13.5%
	Second (2nd semester of 1st year)	162	16.1%
	Third (1st semester of 2nd year)	141	14.0%
	Fourth (2nd semester of 2nd year)	91	9.0%
	Fifth (1st semester of 3rd year)	79	7.8%
	Sixth (2nd semester of 3rd year)	105	10.4%
	Seventh (1st semester of 4th year)	106	10.5%
	Eighth (2nd semester of 4th year)	81	8.0%
	Ninth (1st semester of 5th year)	60	6.0%
	Tenth (2nd semester of 5th year)	46	4.6%
City of residence	Cuenca	248	24.6%
	Guayaquil	189	18.8%
	Quito	570	56.6%

**Table 2:** Faculty and careers description of study participants.

**Faculty**	**Career**	**Quantity**	**Percentage**
Social Sciences and Humanities	Psychology	178	17.7%
	Law	60	6.0%
	Multimedia Design	27	2.7%
	Social Communication	18	1.8%
	Anthropology	2	0.2%
Science and Technology	Architecture	7	0.7%
	Biomedicine	30	3.0%
	Computer Science	43	4.3%
	Electricity	43	4.3%
	Electronics and Automation	24	2.4%
	Automotive Engineering	46	4.6%
	Civil Engineering	77	7.6%
	Industrial Engineering	31	3.1%
	Mechanics	23	2.3%
	Mechatronics	40	4.0%
	Telecommunications	35	3.5%
Administration and Economics	Accounting and Auditing	44	4.4%
	Business Administration	86	8.5%
	Economics	13	1.3%
	Digital Business	5	0.5%
	Management and Leadership	10	1.0%
Life Sciences	Agriculture	2	0.2%
	Biotechnology	60	6.0%
	Nursing	8	0.8%
	Environmental Engineering	28	2.8%
	Veterinary Medicine	15	1.5%
Education	Education	9	0.9%
	Primary Education	18	1.8%
	Early Childhood Education	12	1.2%
	Intercultural Bilingual Education	6	0.6%
	Pedagogy of Physical Activity and Sports	7	0.7%

### 5.2 Instruments

#### 5.2.1 High Five Inventory

Cosentino and Castro Solano (2017) developed a measurement instrument for the factors of the Five Highs model: erudition, tranquility, cheerfulness, honesty, and tenacity. The paper-and-pencil instrument consists of 23 items with scales ranging from 1 (never) to 7 (always). A higher score on each subscale corresponds to a higher level of the respective high factor. High Five Inventory (HFI) exhibited a good fit to the data in both the initial sample (e.g., Comparative Fit Index CFI = 0.968) and the confirmation samples (e.g., CFI = 0.963). The alpha and omega reliabilities for each factor were >0.80.

#### 5.2.2 Academic Motivation Scale

Academic Motivation Scale (AMS) is based on the Self-Determination Theory by Deci and Ryan (2004) and has been adapted for use in Argentina Stover et al. (2012). The dimensions measured by this instrument include extrinsic motivation (external, introjected, identified, and integrated), intrinsic motivation (knowledge-oriented, achievement-oriented, and experience-oriented), and amotivation. The paper-and-pencil instrument comprises 27 items. In applications, it achieved Aiken's coefficients equal to or greater than 0.80.

#### 5.2.3 German Test Anxiety Inventory

Hodapp (1996) developed a measurement instrument known as the German Test Anxiety Inventory (TAI-G), which corresponds to the dual model of Liebert and Morris (1967). It was adapted for use in Argentina by Heredia et al. in 2008. The scales of this instrument include emotionality, worry, cognitive interference, and lack of confidence. The paper-and-pencil inventory consists of 20 items with scales ranging from 1 (never) to 5 (always). A higher score on each scale indicates a higher level of the respective dimension. The TAI-G showed a good fit to the data, with a CFI of 0.97. The alpha and omega reliabilities for each factor exceed 0.80.

#### 5.2.4 Adapted Tuckman Procrastination Scale (ATPS)

Tuckman (1990) developed a measurement instrument known as Adapted Tuckman Procrastination Scale (ATPS), which corresponds to the cognitive and rational-emotive theoretical model of Ellis and Knaus, 1977. It has been adapted for use in Argentina by Furlan et al. (2012). This instrument assesses the procrastination tendencies of university students. The paper-and-pencil scale consists of 16 items with scales ranging from 1 (never happens to me) to 5 (always happens to me). It exhibited a good fit to the data, with a CFI of 0.99. The alpha and omega reliabilities for each factor are >0.80.

#### 5.2.5 Academic performance

Based on the identification numbers of each participating student in the study, the private university was requested to provide the grade point averages achieved in order to have the variable “academic performance.” The criteria for extracting the grades were as follows:

- The number of subjects enrolled and successfully completed during the semester from March to August 2022.
- The specific semester to which the subjects belonged, and the average score obtained for that semester on a scale of 100 points.

### 5.3 Process

The hypotheses were tested using hierarchical multiple linear regression analysis with the Jamovi program (Version 2.4.1) Smadi and Raman (2020). To do this, a link (https://ee.humanitarianresponse.info/x/ieKH1SuM) was provided, containing the instruments along with an invitation to fill them out in the Virtual Learning Cooperative Environments (AVAC, in Spanish) of the Higher Educational Institution. Students from various majors voluntarily responded individually to the instruments via the provided link. Each participant also had the opportunity to review an informed consent form, which included information about the study and a request for authorization and voluntary, anonymous participation in the research. The application took ~15 min to complete due to the number of items in the four psychological tests. In this case, in addition to the self-administered questionnaires, grade point averages for each participant were requested from the private university to obtain the “academic performance” variable.

Data analysis involved hierarchical regression modeling, with the dimensions of Academic Motivation, Test Anxiety, and Academic Procrastination as predictors, and Academic Performance as the outcome variable. Results are presented in terms of multiple linear regression (*R*^2^). The dimensions of one scale are introduced hierarchically within each model, followed by the addition of dimensions from another scale. To assess model significance and variance explanation, a one-way analysis of variance (ANOVA) is employed. The delta (△*R*^2^) is calculated to identify whether the explanation has increased or decreased between the first and second models, and to determine if this difference is statistically significant. An ANOVA test (F) is conducted to determine if there is a significant contribution to variance explanation in predicting academic performance between the first model and the second model (Model 2 vs. Model 1).

## 6 Results

First hypothesis proposed that high factors of the HFM predict academic performance, and the second hypothesis suggested that high factors of the HFM improve the prediction of academic performance compared to the dimensions of academic motivation. In this case, first model includes the seven dimensions of Academic Motivation in relation to academic performance. It is observed that intrinsic motivation oriented toward achievement positively predicts academic performance, while introjected regulation of extrinsic motivation and amotivation negatively predict academic performance.

In the second model, when the Five Highs factors are added, in addition to the variables mentioned above, intrinsic motivation oriented toward knowledge negatively predicts academic performance, and external regulation of extrinsic motivation positively predicts academic performance. Furthermore, the high factors of tranquility and honesty negatively predict academic performance, while tenacity positively predicts academic performance. Notably, the high factor of tenacity emerges as the strongest predictor of academic performance among university students. The second model adds a significant explanation of the variance, as shown in Table 3.

**Table 3:** Multiple linear regression models for hypotheses 1 and 2.

**Predictor**	**Model 1**	**Model 2**
Intrinsic motivation oriented toward stimulating experiences	−0.041	−0.039
Intrinsic motivation oriented toward achievement	0.195^***^	0.143^**^
Intrinsic motivation oriented toward knowledge	−0.095	−0.104^*^
Extrinsic motivation of identified regulation	−0.013	−0.007
Extrinsic motivation of introjected regulation	−0.135^***^	−0.138^***^
Extrinsic motivation of external regulation	0.093	0.106^**^
A motivation	−0.113^***^	−0.101^***^
Erudition		0.014
Peace		−0.084^**^
Joviality		−0.031
Honesty		−0.080^**^
Tenacity		0.228^***^
***R* ^2^**	0.032	0.062
**F**	7.26^***^	8.49^***^
**△*R*^2^**		0.030
**M2 v, 1 F (5 y 1542gl)**		9.93^***^

^*^Indicates a 90% confidence level for the relationship between the outcome and that specific variable. ^**^Denotes 95% confidence ^***^Signifies 99% confidence accordingly.

Regarding the third hypothesis, which posited that the HFM factors enhance the prediction of Academic Performance with respect to dimensions of Test Anxiety, when considered in isolation, it is observed that low self-confidence positively predicts Academic Performance. However, when adding the High Five factors in the second model, Anxiety ceases to be significant, and it is the high factors of peace and honesty that negatively predict Academic Performance, while the high tenacity factor remains the best predictor of Academic Performance. In Model 2, the variance is better explained than in Model 1, as evidenced in Table 4.

**Table 4:** Multiple linear regression models for the hypothesis 3.

**Predictor**	**Model 1**	**Model 2**
Worry	0.005	−0.012
Lack of confidence	0.058^**^	0.015
Emotionality	0.004	−0.031
Interference	−0.048	−0.019
Erudition		−0.009
Peace		−0.097^**^
Joviality		−0.046
Honesty		−0.070^*^
Tenacity		0.235^***^
***R* ^2^**	0.008	0.038
**F**	2.96^**^	6.77^***^
**△*R*^2^**		0.030
**M2 v, 1 F (5 y 1545gl)**		9.75^***^

^*^Indicates a 90% confidence level for the relationship between the outcome and that specific variable. ^**^Denotes 95% confidence. ^***^Signifies 99% confidence accordingly.

Fourth hypothesis posited that the HFM factors enhance the prediction of Academic Performance concerning Academic Procrastination. In the first model, the tendency for procrastination negatively predicts Academic Performance. In the second model, where the HFM factors are added, the procrastination tendency variable becomes non-significant, while the dimensions of peace and honesty negatively predict Academic Performance. Conversely, the high tenacity factor remains the best predictor of Academic Performance. Model 2 provides a significant increase in variance explanation, as observed in Table 5.

**Table 5:** Multiple linear regression models for the hypothesis 4.

**Predictor**	**Model 1**	**Model 2**
Procrastination tendency	−0.070^**^	−0.005
Erudition		−0.002
Peace		−0.085^**^
Joviality		−0.041
Honesty		−0.073^**^
Tenacity		0.238^***^
***R* ^2^**	0.005	0.035
**F**	7.61^**^	9.37^*^
**△*R*^2^**		0.030
**M2 v, 1 F (5 y 1548gl)**		9.68^***^

^*^Indicates a 90% confidence level for the relationship between the outcome and that specific variable. ^**^Denotes 95% confidence. ^***^Signifies 99% confidence accordingly.

Hypothesis five posited that HFM factors improve the prediction of Academic Performance beyond the capacity of common predictors, such as Academic Motivation, Test Anxiety, and Academic Procrastination (hypothesis). Five models are presented: Model 1 represents Academic Procrastination, Model 2 represents Test Anxiety, Model 3 represents Academic Motivation, and Model 4 represents the HFM factors.

In Model 1, it is observed that the procrastination tendency negatively predicts Academic Performance. In Model 2, where Test Anxiety is added, no significant contribution to variance explanation is found. In fact, in this case, the procrastination tendency ceases to be a significant predictor. In Model 3, when Academic Motivation is added, it is noted that one dimension (lack of confidence) of Test Anxiety positively predicts Academic Performance, as well as the dimension of intrinsic achievement-oriented motivation positively predicts. Meanwhile, the dimensions of extrinsic introjected regulation and amotivation negatively predict Academic Performance. Significant variance explanation is added in this case.

Lastly, when the HFM factors are added in Model 4, it is observed that intrinsic achievement-oriented motivation and extrinsic external regulation motivation positively predict Academic Performance, while extrinsic introjected regulation motivation and amotivation negatively predict Academic Performance. Additionally, the high factors of peace and honesty negatively predict Academic Performance, while the high tenacity factor positively predicts Academic Performance. In this case, variance explanation significantly increases. The high tenacity factor remains the best predictor of Academic Performance. Standardized coefficients for the four presented models are displayed in Table 6.

**Table 6:** Multiple linear regression models for the hypothesis 5.

**Predictor**	**Model 1**	**Model 2**	**Model 3**	**Model 4**
Procrastination tendency	−0.070^**^	−0.032	−0.019	0.030
Worry		0.003	−0.008	−0.018
Lack of confidence		0.050	0.066^*^	0.030
Emotionality		0.003	0.022	−0.011
Interference		−0.034	−0.007	0.000
Intrinsic motivation oriented toward stimulating experiences			−0.054	−0.042
Intrinsic motivation oriented toward achievement			0.177^***^	0.143^***^
Intrinsic motivation oriented toward knowledge			−0.100^*^	−0.102^*^
Extrinsic motivation of identified regulation			−0.016	−0.008
Extrinsic motivation of introjected regulation			−0.135^***^	−0.133^***^
Extrinsic motivation of external regulation			0.096	0.107^**^
Amotivation			−0.104^***^	−0.103^***^
Erudition				0.006
Peace				−0.088^**^
Joviality				−0.036
Honesty				−0.078^**^
Tenacity				0.236^***^
***R* ^2^**	0.005	0.008	0.036	0.064
**F**	7.61^**^	2.57^*^	4.77^***^	6.13^***^
**△*R*^2^**		0.003	0.028	0.028
**M2 v, 1 F (4 y 1549gl); M3 v, 2 F (7 y 1542gl); M4 v, 3 F (5 y 1537gl)**		1.31	6.29^***^	9.11^***^

^*^Indicates a 90% confidence level for the relationship between the outcome and that specific variable. ^**^Denotes 95% confidence. ^***^Signifies 99% confidence accordingly.

## 7 Discussion

The overall objective of this study was to determine whether the factors in the HFM predict the academic performance of university students beyond the capacity of common predictors, such as academic motivation, test anxiety, and academic procrastination. To achieve this, a predictive study was conducted to test the proposed theoretical model through the analysis of linear regressions of the High Five model factors as predictors of academic performance, while considering common predictors like academic motivation, test anxiety, and academic procrastination. The following discussion will cover the results presented in previous sections, along with insights from other empirical studies in the field.

Regarding the findings regarding the prediction of academic performance by HFM factors and their ability to improve the prediction of academic performance with respect to dimensions of academic motivation, it is observed that both introjected extrinsic regulation motivation and amotivation negatively predict academic performance. These results align with the work of Baker (2004), Deng and Shi (2023), Petersen et al. (2009), Sommer and Dumont (2011), who consider that both extrinsic motivation and amotivation negatively predict academic performance.

Furthermore, the data from the analysis reflect that intrinsic motivation oriented toward achievement positively predicts academic performance, which is in line with the work of Baker (2004), Bailey and Phillips (2016), who assert that intrinsic motivation is the best predictor of academic performance. However, when the HFM factors were added to the statistical analysis, it is presented that the dimension of intrinsic motivation oriented toward knowledge negatively predicts academic performance, a result in line with the work of Baker (2004), Bailey and Phillips (2016), who state that intrinsic motivation oriented toward knowledge is the only significant predictor of academic performance. Additionally, the evidence found in the study suggests that the dimension of extrinsic external regulation motivation positively predicts academic performance, a result consistent with the work of Deng and Shi (2023), who argue that extrinsic external regulation motivation significantly predicted academic performance.

The evidence reported shows that the high factors of peace and honesty negatively predict academic performance, which is not consistent with the work of Castro Solano and Cosentino (2019), who claim that the high factors of tenacity and erudition were the only ones positively associated with academic performance. The differences found in this study can be explained by two reasons: first, the objective measurement of academic performance, and second, because the high factor of peace represents positive traits related to patience, tolerance, calmness, and serenity, while the high factor of honesty represents positive traits related to loyalty, trust, values, transparency, and veracity, as explained by Cosentino and Castro Solano (2017). Therefore, lower values in the high peace factor and high honesty factor will have a more negative impact on academic performance.

Furthermore, the findings from the statistical analysis demonstrate that the high tenacity factor positively predicts academic performance, which is consistent with the work of Castro Solano and Cosentino (2019), who indicate that the high tenacity factor positively predicts academic performance. This result is also supported by other studies such as those by Sorić et al. (2017), Stajkovic et al. (2018), Vedel (2014), Hidalgo-Fuentes et al. (2021), Wagerman and Funder (2007), Poropat (2014), Duff et al. (2004), De Feyter et al. (2012), O'Connor and Paunonen (2007), who assert that the personality trait of conscientiousness (referred to as tenacity in the HFM) is the only predictor of academic performance.

Results of linear regression analysis confirm hypotheses 1 and 2 of this study, which posited that the HFM factors predict academic performance and improve the prediction of academic performance with respect to dimensions of academic motivation.

The HFM factors can enhance the prediction of academic performance in relation to dimensions of test anxiety. When analyzing Test Anxiety independently, it is observed that a lack of confidence is positively correlated with academic performance. However, this finding contrasts with the study by Piemontesi and Heredia (2011), where a lack of confidence had a weak negative correlation with academic performance. The differences found in this study can be explained by two reasons: first, the objective measurement of academic performance, and second, because university students exhibit self-efficacy related to their belief in being able to control environmental demands (Hodapp et al., 1995). However, when the HFM factors were included in the statistical analysis, Test Anxiety ceases to be significant. Instead, the high factors of peace and honesty negatively predict academic performance, which is not consistent with the work of Castro Solano and Cosentino (2019), who claimed that the high factors of tenacity and erudition were the only ones positively associated with academic performance. The differences found in this study can be explained by two reasons: first, the objective measurement of academic performance, and second, because the high peace factor represents positive traits related to patience, tolerance, calmness, and serenity, while the high honesty factor represents positive traits related to loyalty, trust, values, transparency, and veracity as explained by Cosentino and Castro Solano (2017). Therefore, lower values in the high peace factor will have a more negative impact on academic performance, which is also observed in the case of the high honesty factor.

Additionally, the findings from the statistical analysis show that the high tenacity factor positively predicts academic performance, which is consistent with the findings of Castro Castro Solano and Cosentino (2019), Duff et al. (2004), De Feyter et al. (2012), Hidalgo-Fuentes et al. (2021), O'Connor and Paunonen (2007), Poropat (2014), Sorić et al. (2017), Stajkovic et al. (2018), Vedel (2014), Wagerman and Funder (2007), who state that the personality trait of conscientiousness (referred to as tenacity in the MCA) is the only predictor of academic performance.

In summary, the results of the linear regression analysis once again confirm hypotheses 1 and 3, which posited that the HFM factors would improve the prediction of academic performance with respect to dimensions of test anxiety.

Meanwhile, concerning whether the HFM factors would be capable of improving the prediction of academic performance with respect to academic procrastination, it is found that the procrastination tendency negatively predicts academic performance. These results are consistent with the work of Furlan et al. (2012), Lamas (2015), Kim and Seo (2015), Jones and Blankenship (2021), who revealed that the procrastination tendency is negatively correlated with academic performance. However, when the HFM factors are added to the statistical analysis, it is presented that the procrastination tendency ceases to be significant. Instead, the high factors of peace and honesty negatively predict academic performance, which is not consistent with the work of Castro Solano and Cosentino (2019), who claimed that the high factors of tenacity and erudition were the only ones positively associated with academic performance. The differences found in this study can be explained by two reasons: first, the objective measurement of academic performance, and second, because the high peace factor represents positive traits related to patience, tolerance, calmness, and serenity, while the high honesty factor represents positive traits related to loyalty, trust, values, transparency, and veracity as explained by Cosentino and Castro Solano (2017). Therefore, lower values in the high peace factor will have a more negative impact on academic performance, which is also observed in the case of the high honesty factor.

Furthermore, the findings from the analysis show that the high tenacity factor positively predicts academic performance, which is consistent with the work of Castro Solano and Cosentino (2019), who consider that the high tenacity factor positively predicts academic performance. This result is also supported by other studies, such as those by Duff et al. (2004), De Feyter et al. (2012), Hidalgo-Fuentes et al. (2021), Poropat (2014), O'Connor and Paunonen (2007), Sorić et al. (2017), Stajkovic et al. (2018), Vedel (2014), Wagerman and Funder (2007), who assert that the personality trait of conscientiousness (referred to as tenacity in the HFM) is the only predictor of academic performance.

Results of linear regression analysis once again confirm hypotheses 1 and 4, as the HFM factors have predicted academic performance and academic procrastination.

Regarding whether the HFM factors can improve the prediction of academic performance beyond the capacity of typical predictors such as academic motivation, test anxiety, and academic procrastination, it is found that when the procrastination tendency is added to the statistical analysis, it negatively predicts academic performance. This finding is consistent with the work of Furlan (2013), Jones and Blankenship (2021), Kim and Seo (2015), Lamas (2015), Ryan and Deci (2000), who have asserted the existence of a negative association between procrastination tendency and academic performance.

Then, when the variable test anxiety is added to the statistical analysis, it is observed that it does not significantly contribute to explaining the variance. Consequently, the procrastination tendency ceases to be a significant predictor.

Additionally, when academic motivation is included in the analysis, it is noted that one dimension, namely, lack of confidence, from the test anxiety scale positively predicts academic performance. This result is not consistent with the work of Piemontesi and Heredia (2011), who stated that lack of confidence was negatively but weakly correlated with academic performance. The differences found in this study can be attributed to two possible reasons: first, the objective measurement of academic performance, and second, university students exhibit self-efficacy related to the belief in their ability to control environmental demands (Hodapp et al., 1995).

Furthermore, intrinsic motivation oriented toward achievement positively predicts academic performance, which aligns with the findings of Baker (2004), Deng and Shi (2023), Bailey and Phillips (2016), Sommer and Dumont (2011), who argued that intrinsic motivation is the only significant predictor of academic performance.

Additionally, the analysis reveals that the dimension of extrinsic motivation of introjected regulation negatively predicts academic performance. This finding is in line with the work of Petersen et al. (2009), who found a negative prediction of extrinsic motivation on academic performance.

Lastly, it is found in the statistical analysis that amotivation negatively predicts academic performance, which is consistent with the findings of Baker (2004), Deng and Shi (2023), Bailey and Phillips (2016), Sommer and Dumont (2011), who stated that amotivation negatively predicts academic performance.

However, when the HFM factors are added to the statistical analysis, it is observed that extrinsic regulation of external motivation positively predicts academic performance, which is consistent with the work of Sommer and Dumont (2011). In addition, it is found in the analysis that intrinsic motivation oriented toward achievement positively predicts academic performance, which aligns with the findings of Baker (2004), Deng and Shi (2023), Bailey and Phillips (2016), Sommer and Dumont (2011), who argued that intrinsic motivation is the only significant predictor of academic performance. Furthermore, it is found in the statistical analysis that introjected regulation of extrinsic motivation negatively predicts academic performance, which aligns with the findings of Deng and Shi (2023). Additionally, it is found in the statistical analysis that amotivation negatively predicts academic performance, which is consistent with the findings of Baker (2004), Deng and Shi (2023), Bailey and Phillips (2016), Sommer and Dumont (2011).

However, when the Five Highs are added to the statistical analysis as the sole predictor of academic performance, without including anything else other than the Five Highs factors, it is found that the high factors of peace and honesty negatively predict academic performance. This finding is not in line with the work of Castro Solano and Cosentino (2019), where it is suggested that the high factors of tenacity and erudition were the only ones positively associated with academic performance. The differences found in this study can be explained by two possible reasons: first, the objective measurement of academic performance, and second, because the high peace factor is the positive trait of patience, tolerance, tranquility, and serenity, and the high honesty factor is the positive trait of loyalty, trust, values, transparency, and veracity as outlined by Cosentino and Castro Solano (2017). Therefore, the lower the value of the high peace factor, the more negatively it affects academic performance, and the same holds for the high honesty factor.

At the same time, our analysis found that the high tenacity factor positively predicts academic performance. These findings are supported by the work of Castro Solano and Cosentino (2019), Duff et al. (2004), De Feyter et al. (2012), Hidalgo-Fuentes et al. (2021), O'Connor and Paunonen (2007), Poropat (2014), Sorić et al. (2017), Stajkovic et al. (2018), Vedel (2014), Wagerman and Funder (2007), who state that the personality trait of responsibility (referred to as tenacity in the HFM) is the only predictor of academic performance.

The HFM is not only related to academic performance but also to common psychological predictors such as academic motivation, test anxiety, and academic procrastination. These factors, in turn, are linked to academic performance. Despite the stable personality traits detected by the high factors of Academic Motivation, in contrast to the stable personality traits detected by the factors of the Big Five model (Goldberg, 1981; Vallerand et al., 1992; Komarraju et al., 2009; Francisquelo and Furlan, 2015; Diav et al., 2017; Saraswati, 2017; Curelaru and Diac, 2021; Börekci, 2022; Ocansey et al., 2022), the HFM has proven to be a viable and suitable model. Previous studies, such as those by Castro Solano and Cosentino (2019), Cosentino and Castro Solano (2017), have pointed out that the high factors are reasonably linked to many variables and, with the findings of the present research, also establish a precedent that contributes practical value in Educational Psychology and Positive Psychology.

It is important to highlight that the variable of prior academic performance was not included in the linear regression analysis in the present study, despite being one of the best predictors of academic performance. Research by authors such as Eysenck (1981), Furnham (1992), Busato et al. (1998) raises concerns that previous academic performance as a composite variable addresses various factors that can make the outcome unpredictable (such as the student's effort in their academic experience prior to university enrollment, their grades, attitude, will, effort, and teaching characteristics received) TEJEDOR (2003). There is empirical evidence that personality is the best predictor of academic performance (Eysenck, 1981; Furnham, 1992; Busato et al., 1998).

Other authors assert that personality measures by themselves are powerful enough to explain a moderate percentage of the variation in academic performance (Wolfe and Johnson, 1995; Blickle, 1996; Cacioppo et al., 1996; De Raad and Schouwenburg, 1996; Rindermann and Neubauer, 2001; Chamorro-Premuzic and Furnham, 2003; Ackerman et al., 2011), although some earlier studies, in particular Wolfe and Johnson (1995), Vroom (1960), Chorro (1981), Hamilton and Freeman (1971), had claimed this relationship long before. Hence, for the purpose of this study, it was crucial to demonstrate that the Five Highs model can predict academic performance solely with the HFM factors, without considering other models known to be linked to academic performance. Therefore, to enhance the statistical analysis of academic performance, objective measures were employed, such as institutional record grades.

Hence, the results of the linear regression analysis once again confirm hypotheses 1 and 5, as the high factors of HFM predict academic performance beyond the capacity of typical predictors, such as academic motivation, test anxiety, and academic procrastination.

While the *R*^2^ values in our regression analysis are relatively low, we believe there are robust arguments to justify the relevance of our findings. Firstly, it is essential to highlight the magnitude of our sample, which includes data from 1,532 university students. This large sample provides us with significant statistical power to detect even small relationships between variables. Furthermore, by utilizing real average data on academic performance, we are working with concrete and reliable measures that reflect the reality of students.

Secondly, academic performance is an extremely complex and multifactorial variable. Countless tangible and intangible factors can influence student performance. In this context, it is natural that any regression model explains only a portion of the variability. Our study does not claim to be a definitive solution but rather an important step toward better understanding the relationships between the analyzed factors and academic performance.

Moreover, the fact that our predictors, including high HFM factors, exhibit statistically significant relationships with academic performance indicates a foundation for future research and potential interventions. Despite the low *R*^2^ values, we have identified relationships that could be of great importance to the university and the academic community.

Similarly, when aiming for a perfect fit in multiple linear regression analysis, several studies demonstrate that, in order to estimate potential relationships between personality factors and other variables, a small sample is required to achieve an *R*^2^ value of 1.0 for a perfect fit. There is supporting evidence for this assertion. In a study conducted by Corte de la Corte et al. (2016) to analyze the predictive role of different types of aggression in peer relationships and the moderating effect of certain personality variables, with a sample size of 54 individuals, they found a goodness of fit in the conscientiousness factor with an *R*^2^ value of 0.298.

In the other hand, Delhom et al. (2019) investigated how personality traits are associated with six dimensions of psychological wellbeing in a sample of 224 elderly Spaniards, finding a goodness of fit in the neuroticism factor with an *R*^2^ value of 0.351, indicating a perfect fit. Finally, Arias et al. (2020) in their research to assess the influence of character strengths on psychoeducational variables such as Academic Resilient Personality (ARP) and Academic Engagement (AE), in a sample of 263 university students, found a goodness of fit in the correlation between ARP and AE with an *R*^2^ value of 0.531. Therefore, the present study has a much larger sample size that could impact the perfect fit of the *R*^2^.

Ultimately, analyses revealed that only the variables Erudition and Tenacity exhibited considerable multicollinearity, with Variance Inflation Factor (VIF) of 24.34 and 21.65, respectively. Despite these high values, the decision was made not to exclude these variables from our models. This choice is justified by their theoretical relevance and the need to maintain the integrity of the constructs being examined.

Our goal is to contribute to knowledge and provide relevant information that can be useful for making informed decisions in the academic sphere. While *R*^2^ values may be low, we believe the obtained results are valuable in the context of continuous improvement in students' academic outcomes.

The new model is valuable as it provides insights to Educational Psychology and Positive Psychology by offering a broader conceptual network of the HFM to relate them to academic performance, aiming to push the boundaries of knowledge in these areas. Simultaneously, the need to integrate new theoretical studies, both theoretically and practically, helps to solidify knowledge and gain experiences regarding the Five Highs model in different contexts.

These models inclusions for predicting academic outcomes allows us to accurately forecast future results, which is useful to consider in the educational institutions assessments, whether at admission or throughout the students' academic trajectory.

Finally, institutions must raise awareness that positive personality traits should be nurtured among students. It is imperative for the institution to conduct intervention workshops to reinforce the positive characteristics that underpin good academic outcomes.

## 8 Conclusions

In the present study, it is concluded that, firstly, the high factors of HFM predict academic performance. Specifically, the high factors of HFM, honesty, and peace predict academic performance negatively, while the high factor tenacity predicts academic performance positively among university students.

Secondly, the high factors of HFM enhance the prediction of academic performance compared to dimensions of Academic Motivation. When added as the sole predictor of HFM in the statistical analysis, apart from Academic Motivation, the dimension of intrinsic motivation oriented toward knowledge predicts academic performance negatively, and extrinsic motivation of external regulation predicts academic performance positively. Additionally, the high factors of HFM, peace, and honesty, predict academic performance negatively, while the high factor tenacity predicts academic performance positively among university students.

Thirdly, the high factors of Academic Motivation improve the prediction of academic performance compared to dimensions of Test Anxiety. When added as the sole predictor of academic performance in the statistical analysis, apart from Test Anxiety, anxiety ceases to be significant. In this context, the high factors of Academic Motivation, peace, and honesty, predict academic performance negatively, while the high factor tenacity predicts academic performance positively among university students.

Fourthly, the high factors of HFM enhance the prediction of academic performance with regard to procrastination tendencies. When added as the sole predictor of academic performance in the analysis, apart from procrastination tendencies, they cease to be significant. In this scenario, the high factors of HFM, peace, and honesty, predict academic performance negatively, while the high factor tenacity serves as a positive predictor of academic performance among university students.

Fifthly, the high factors of HFM improve the prediction of academic performance with respect to dimensions of Academic Motivation, Test Anxiety, and Academic Procrastination. When added as the sole predictor of HFM in the analysis, apart from the usual psychological predictors, it is observed that intrinsic motivation oriented toward achievement and extrinsic motivation of external regulation predict academic performance positively. Meanwhile, intrinsic motivation of introjected regulation and amotivation predict academic performance negatively. Additionally, the high factors of HFM, peace, and honesty, predict academic performance negatively, while the high factor tenacity predicts academic performance positively.

This study demonstrates that the HFM can extend beyond the typical psychological predictive models associated with academic performance. Furthermore, through this statistical analysis, it succeeds in expanding the field, generating a broader conceptual framework for the Academic Motivation model.

One possible limitation of the research is that study was cross-sectional. It would be interesting to design a longitudinal study because quality control is essential over time, allowing us to measure the effect and exposure of high HFM factors at different time points. Additionally, longitudinal analyses can be conducted within the context of generalized linear models as a conventional regression tool to relate the effect to different exposures and consider the correlation of measures between subjects (González Mares, 2019). Another limitation to consider is the lack of sample randomization. It would be interesting to use this technique to balance the effect of external or uncontrollable conditions that may influence the results.

Cultural considerations cannot be considered limitations for personality research. McCrae and Costa (2003) examined the applicability of the factors of the BFM in a wide variety of cultures, finding that there is much more to personality than just traits, but the traits identified in the factors of the BFM seem to offer a solid cross-cultural foundation for understanding personality worldwide. It is true that there are differences in personality traits among cultural groups, but variability also appears within a specific culture (McCrae et al., 2005).

Future studies may consider including the prior academic performance of university students in the statistical analysis. Similarly, it would be beneficial to investigate the comparison between the BFM and the HFM to demonstrate that the traits of the Academic Motivation model are as stable as the traits of the BFM.

Finally, it is suggested that future studies utilize a completely random sample for more robust results.

## Data availability statement

The raw data supporting the conclusions of this article will be made available by the authors, without undue reservation.

## Ethics statement

Ethical approval was not required for the studies involving humans because the study was in compliance with institutional policies, had voluntary participation and was judged to be low-risk research. The studies were conducted in accordance with the local legislation and institutional requirements. The participants provided their written informed consent to participate in this study.

## Author contributions

JQ-C: Writing – review & editing, Writing – original draft, Visualization, Validation, Supervision, Resources, Methodology, Investigation, Formal analysis, Data curation, Conceptualization. AC: Writing – review & editing, Writing – original draft, Validation, Methodology, Data curation, Conceptualization.
